# Ketamine and the Disinhibition Hypothesis: Neurotrophic Factor-Mediated Treatment of Depression

**DOI:** 10.3390/ph16050742

**Published:** 2023-05-12

**Authors:** Philip Borsellino, Reese I. Krider, Deanna Chea, Ryan Grinnell, Thomas A. Vida

**Affiliations:** Kirk Kerkorian School of Medicine at UNLV, 625 Shadow Lane, Las Vegas, NV 89106, USA; borsep1@unlv.nevada.edu (P.B.); krider1@unlv.nevada.edu (R.I.K.); grinnr1@unlv.nevada.edu (R.G.)

**Keywords:** ketamine, esketamine, arketamine, major depressive disorder, treatment-resistant depression, brain-derived growth factor, synaptogenesis

## Abstract

Ketamine is a promising alternative to traditional pharmacotherapies for major depressive disorder, treatment-resistant depression, and other psychiatric conditions that heavily contribute to the global disease burden. In contrast to the current standard of care medications for these disorders, ketamine offers rapid onset, enduring clinical efficacy, and unique therapeutic potential for use in acute, psychiatric emergencies. This narrative presents an alternative framework for understanding depression, as mounting evidence supports a neuronal atrophy and synaptic disconnection theory, rather than the prevailing monoamine depletion hypothesis. In this context, we describe ketamine, its enantiomers, and various metabolites in a range of mechanistic actions through multiple converging pathways, including N-methyl-D-aspartate receptor (NMDAR) inhibition and the enhancement of glutamatergic signaling. We describe the disinhibition hypothesis, which posits that ketamine’s pharmacological action ultimately results in excitatory cortical disinhibition, causing the release of neurotrophic factors, the most important of which is brain-derived neurotrophic factor (BDNF). BDNF-mediated signaling along with vascular endothelial growth factor (VEGF) and insulin-like growth factor 1 (IGF-1) subsequently give rise to the repair of neuro-structural abnormalities in patients with depressive disorders. Ketamine’s efficacious amelioration of treatment-resistant depression is revolutionizing psychiatric treatment and opening up fresh vistas for understanding the underlying causes of mental illness.

## 1. Introduction

Major depressive disorder (MDD) is one of the most prevalent diseases in the world, affecting around 280 million individuals as of 2019 [1]. In the US alone, MDD is overabundant, affecting an estimated 21 million adults in 2020 [2]. MDD can be a debilitating diagnosis that often presents with symptoms such as anhedonia, insomnia, fatigue, and feelings of worthlessness, according to the Diagnostic and Statistical Manual of Mental Disorders (DSM-5) [3]. MDD is also a major burden on health and can lead to poor quality of life, impaired functioning, and increased risk of suicidality [4,5]. The current standard of care treatments for MDD include pharmacotherapeutics such as selective serotonin reuptake inhibitors (SSRIs) [6] or serotonin-norepinephrine reuptake inhibitors (SNRIs) [7], adjunctive or primary psychotherapy [8], and somatic treatments such as electroconvulsive therapy [9]. While these therapies prove effective for many patients and are more efficacious than a placebo for both initial therapy and maintenance treatment, many more patients suffer from MDD that is resistant to treatment [6,10]. The failure of at least two different antidepressant therapeutics in the treatment of MDD classifies as treatment-resistant depression (TRD) [11]. Further, current pharmacological interventions have the drawback of a slow onset of action, with therapeutic efficacy occurring on the order of weeks to months [12]. Even after therapeutic effects initiate, current pharmacological interventions require long-term administration for the maintenance of therapeutic efficacy [13].

Improvements are needed in the efficacy of the pharmacological treatment of MDD, the onset of action, and the sustainability of response and remission [14]. With the rising prevalence of MDD in the US, treatment-resistant depression (TRD) is also occurring at an increased rate [15]. In a study synthesizing adult claims of Medicare, Medicaid, commercial plans, and the US Veterans Health Administration (VHA), an estimated 8.9 million adults were treated for MDD over twelve months in 2017, and 2.8 million (30.9%) were diagnosed with TRD [16]. The social and fiscal burden that accompanies the increased prevalence of TRD suggests that more effective treatments and a more complete understanding of the underlying mechanisms of depression are desperately needed.

The prevailing mechanistic explanation of MDD has shifted in recent years, as burgeoning research challenges the previously accepted model of monoamine depletion. Depletion in neurotransmitter levels with serotonin, norepinephrine, and/or dopamine as the root cause for MDD (the monoamine hypothesis of depression) has been favored since the 1990s [17]. This cellular and molecular model specifically focuses on serotonin (5-hydroxytryptamine or 5-HT) and, in some cases, norepinephrine, and the various mechanisms by which they are lacking at the synaptic cleft. This can occur due to the overexpression of the serotonin transporter, SERT, removing the neurotransmitter from the presynaptic cleft, or lowering its precursor, tryptophan, causing inadequate serotonin production [18]. Similar defects can occur with the norepinephrine transport, NET [19]. This general mechanism of action is the target of many SSRIs and SNRIs to maintain serotonin or norepinephrine in the presynaptic cleft for a longer duration [20,21].

The traditional treatment for MDD relies heavily on the monoamine theory of depression [18]. However, SSRIs and related therapies have relatively low efficacy (30–50% treatment resistance) and delayed clinical outcomes, often requiring many weeks of treatment before seeing any clinical benefit [22]. Furthermore, the large prevalence of TRD argues against the efficacy of traditional medications. Recently, several systematic and narrative reviews focused on the efficacy of the monoamine hypothesis and revealed the inconsistent consequences of serotonin, norepinephrine, or dopamine depletion [18,23,24]. Much research in support of the serotonin hypothesis fails to account for the risk of bias when interpreting information, does not reach a statistical significance of *p* ≤ 0.05, or includes the prior use of antidepressants in participants that skewed therapeutic outcomes [18]. In addition to the weak clinical evidence in support of the serotonergic model, this framework does not account for structural brain changes during chronic MDD and TRD. Altered brain structure, including the reduced volume of the prefrontal cortex and hippocampus, and the deterioration of neuronal connections, occurs in MDD and TRD [25]. A newer view posits that neuronal atrophy including dendritic spine loss and impaired synapse formation is the underlying basis of depression and other mood disorders [24,26]. Glutamate neurotransmission plays a key role in these mechanisms [27]. For example, metabotropic glutamate receptors (mGluRs) in the hippocampus are the targets for synaptic plasticity or neuronal growth and show robust, antidepressant effects when activating negative allosteric modulators (NAMs) of mGlu2 and mGlu3 receptors [28]. These effects occur with N-methyl-D-aspartate receptor (NMDAR) antagonism, leading to mGluR activity and a glutamate surge. In addition, increasing the expression of brain-derived neurotrophic factor (BDNF) and vascular endothelial growth factor (VEGF) may contribute to the maintenance and number of synapses in hippocampal neuritogenesis [29]. Studies on the mechanism of action for the fast-acting antidepressant, ketamine, have greatly informed this newer neurotrophic view of depression [26,30].

## 2. Review

### 2.1. Ketamine’s Unique Pharmacological Properties

Ketamine was first synthesized as CI-581 at Parke-Davis & Co. Detroit, MI, USA (now a subsidiary of Pfizer) [31] in an attempt to create a short-acting derivative of a related compound, phencyclidine (PCP). PCP was not useful as an anesthetic in the previous decade, owing in large part to its psychotomimetic properties [32]. Ketamine was shown to have dissociative and anesthetic properties, with less pronounced psychotomimetic effects [33], and was initially introduced as a short-acting anesthetic in 1970 (for a historical review see [31,34,35,36]). It had been anecdotally described as an antidepressant since the late 1970s [31], but it was not until 2000 that this relationship was studied in the first human clinical trial [37]. Since then, many studies have provided evidence not only of antidepressant effects [38,39], but also of a reduction in suicidality [40,41]. The US Food and Drug Administration approved (*S*)-ketamine for therapy in treatment-resistant depression in 2019 as the nasal spray Spravato™ [42]. Furthermore, ketamine shows therapeutic antidepressant effects in bipolar depression [43,44], bipolar disorder [45], and treatment-resistant bipolar disorder [46]. Overall, ketamine therapy offers a rapid-acting reversal (in hours) of depressive symptoms and has opened up an exciting new frontier for the treatment of MDD and related disorders [35,47,48,49].

Ketamine (IUPAC name: 2-(2-chlorophenyl)-2-(methylamino)cyclohexan-1-one) [50] is classified as a cyclohexanone and as a monochlorobenzene [51,52]. Chlorobenzenes are ketamine’s direct parent, in which one or more chlorine atoms are attached to a benzene; in ketamine’s case, only one is attached to the benzene ring [51,53]. PCP is considered a piperidine rather than a chlorobenzene, but both agents share the same aromatic heteromonocyclic molecular framework [53]. Additionally, PCP’s direct molecular parent is the same as one of ketamine’s alternative parents, aralkylamines [51]. Other similar molecules include methoxetamine, a drug that is also experiencing a revival due to its potential antidepressant effects, and eticyclidine, a member of the human exposome with unknown significance [54,55]. Classically, ketamine is considered mainly an N-methyl-D-aspartate receptor (NMDAR) antagonist [56,57], but newer evidence has revealed NMDAR-independent mechanisms as a part of its antidepressant action [58,59]. Further, ketamine has additional receptor or enzyme targets, as presented in Table 1.

Ketamine’s mechanism of action as an anesthetic agent relies on its non-competitive antagonism of the NMDAR. These anesthetic effects, coupled with maintained blood pressure, spontaneous respiration, and laryngeal reflexes, made it a useful agent in various clinical scenarios [69]. Both ketamine and PCP act as NMDAR antagonists, though ketamine is a less potent NMDAR antagonist and produces fewer psychiatric side effects [53,70]. However, ketamine still has similar anesthetic and analgesic activity, making it an excellent replacement for short-lived trials of PCP as an anesthetic [70]. Other pertinent NMDAR antagonists include dextromethorphan, tramadol, methadone, and agmatine, all of which have significantly different effects on the CNS system and are therapeutically distinct [53].

### 2.2. Ketamine and Its Metabolites Show Differences in Pharmacology and Antidepressant Effects

**Figure 1:**
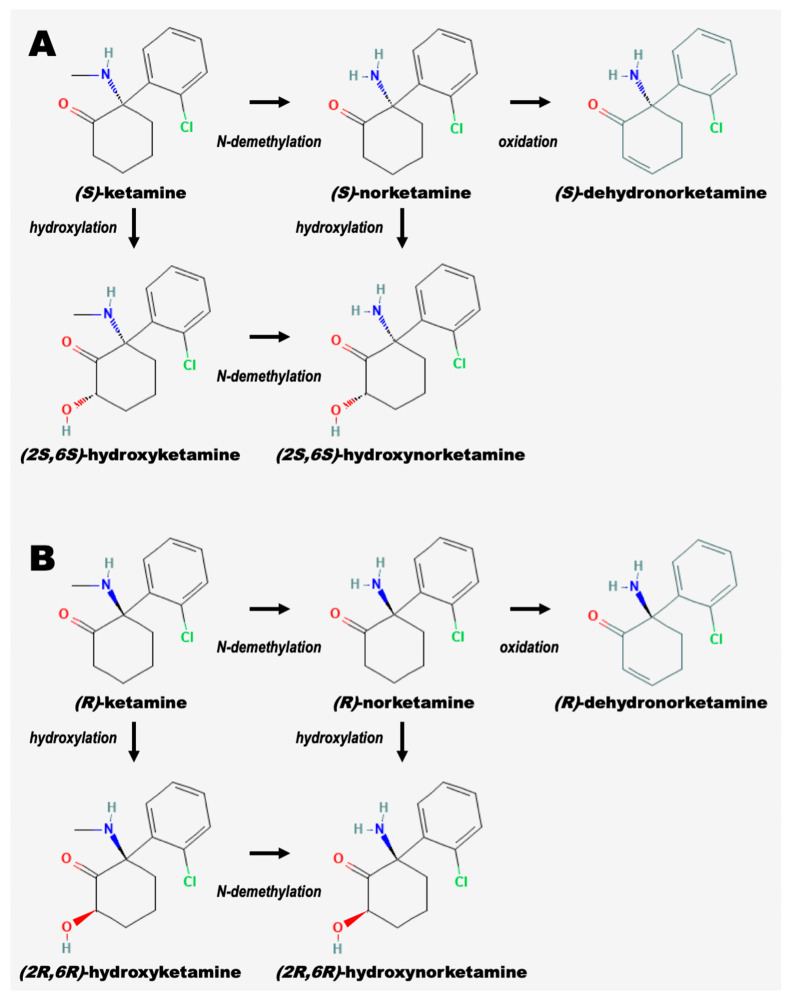
**Intermediates in the metabolism of ketamine enantiomers.** Cytochrome P450 (CYP) enzymes in the liver N-demethylate ketamine or hydroxyketamine to produce norketamine or hydroxynorketamine, respectively. The oxidation of norketamine to produce dehydronorketamine also occurs. CYP enzymes can hydroxylate ketamine and norketamine to produce hydroxyketamine and hydroxynorketamine, respectively. (**A**) depicts the metabolism of (*S*)-ketamine (esketamine), while (**B**) depicts the metabolism of (*R*)-ketamine (arketamine). All structures are derived from the PubChem database (https://pubchem.ncbi.nlm.nih.gov/, accessed on 3 May 2023). The overall figure is adapted from [72,73].

Ketamine is metabolized through the hepatic system, initially characterized by N-demethylation to norketamine [71,72,73]. Subsequent reactions include hydroxylation of the cyclohexone ring, conjugation to glucuronic acid, and dehydration until ultimately clearing the body primarily in the urine [53,74]. CYP3A4 and CYP2B6 are the primary liver enzymes responsible for ketamine metabolism. CYP3A4 metabolizes (*S*)-ketamine faster than (*R*)-ketamine. CYP2B6 metabolism rates are equal between the two enantiomers [75]. Interestingly, doses of racemic ketamine inhibit the elimination of (*S*)-ketamine, indicating that (*R*)-ketamine affects the metabolic clearance of its enantiomeric partner [76]. Ketamine is absorbed, distributed, and cleared rapidly. Intramuscular injection shows a bioavailability of around 93%, but oral dosage only results in 17% bioavailability, due to extensive first-pass metabolism [77]. Ketamine boasts an impressive distribution half-life of around 1.95 min [77]. With an elimination time of only 186 min and a clearance rate of 95 L/h/70 kg (±13%), ketamine and its metabolites’ quick excretion have only garnered additional interest for its rapid antidepressant mechanisms [74,78,79]. The pathways for metabolism of the (*R*) and (*S*) ketamine enantiomers with intermediates are depicted in Figure 1.

Clinically, ketamine has traditionally been administered as a racemic mixture, with each enantiomer having different therapeutic effects. In addition to the two enantiomers, ketamine’s metabolites have been implicated in its therapeutic effectiveness [58,78,80]. Of ketamine’s metabolites, norketamine and specifically (*2R*,*6R*)-hydroxynorketamine (HNK) hold the most clinical relevance. These metabolites are detected in mouse brain tissue within 10 min of ketamine administration [58,78]. Recent work examined HNKs and their pharmacological relevance [81,82]. Most notably, (*2R*,*6R*)-HNK shows potential therapeutic efficacy at a dose that does not involve the direct inhibition of the NMDAR receptor [82,83,84] (see below).

Ketamine’s established association with several acute side effects is a significant factor in the search for safer yet clinically effective enantiomers. For example, administering ketamine to patients with psychotic disorders can exacerbate both positive and negative schizophrenic symptoms [85]. In patients with TRD, the side effects of ketamine treatment are mostly acute, transient, and self-resolving [86]. The most commonly reported acute side effects are headache, dizziness, disassociation, anxiety, temporary hypertension, and blurry vision [86]. However, research on chronic side effects is lacking, which is concerning for patients with TRD that might require long-term use. In the context of recreational use, repeated high doses can lead to urogenital dysfunction through urothelial apoptosis, hepatotoxicity, cognitive deficits in both short and long-term memory, and dependency [86]. Of note, recreational doses and the subsequent side effects can differ from those found in clinical use. For example, one year of esketamine intranasal treatment was not reported to result in cognitive impairment [87] and has not been found to increase the risk of new-onset drug abuse. Nonetheless, 90.1% of patients in the SUSTAIN-2 trial [88] experienced at least one treatment-emergent adverse event. Additionally, the side effects found throughout one year of the trial were consistent with the side effects reported in short-term studies [88].

#### 2.2.1. Arketamine

The (*R*) enantiomer of ketamine, arketamine, has garnered interest for its therapeutic potential, with a relative lack of dissociative effects. An open-label pilot study demonstrates that arketamine has similar efficacy for treatment-resistant depression compared to esketamine [72,89]. In both animal and human trials, a single arketamine dose has been associated with fewer adverse side effects than racemic ketamine, possibly due to its lower NMDAR activity compared to esketamine [72,89]. The differences between arketamine and esketamine in binding affinities, particularly with regards to the NMDAR, make it a compelling and interesting candidate for antidepressant therapy, as it has fewer side effects than esketamine [72,90].

Although its precise mechanism of action for antidepressant efficacy has yet to be determined, many proposed mechanisms revolve around neurotropic growth factors (see below). Arketamine has a unique ability to stimulate BDNF release through an NMDAR-independent mechanism. Arketamine also induces TGF-β1 expression and the subsequent release in microglia activating the BDNF-TrkB pathway in a nearby neuron and leading to synaptogenesis [72].

#### 2.2.2. Esketamine

Esketamine is the predominant enantiomer responsible for its anesthetic and analgesic activities despite FDA approval for depressive therapy [75]. Esketamine is cleared faster and has more psychotomimetic effects compared to arketamine [71,90]. The (*S*) enantiomer binds strongest to the NMDA receptor and has a two to four-time greater potency for analgesic effects with similar ratios for the opioid and muscarinic receptors [62,67,79,90]. The greater affinity for the classic ketamine receptors also leads to increased side effects. However, esketamine has a proven efficacy for antidepressant treatment, and the recent searches for other effective metabolites are efforts to avoid unwanted side effects and abuse potential [75].

NMDAR inhibition largely underlies the therapeutic mechanism of esketamine [91]. In a double-blind RCT, intranasal esketamine in conjunction with a traditional oral antidepressant resulted in greater and quicker improvements in treatment-resistant depression [91]. In terms of adverse side effects, 7% of the treatment group had to discontinue the study, but most adverse events resolved within 1.5 h [91]. Eukaryotic elongation factor 2 kinase (eEF2K) has been implicated in ketamine’s antidepressant effects, and its activation through a cascade started by NMDAR inhibition lends itself to being more effective with esketamine [92]. Interestingly, this pathway leads to BDNF release, converging with one of arketamine’s proposed mechanisms of antidepressant action (see below).

#### 2.2.3. Norketamine

Norketamine is a primary metabolite of ketamine [71]. (*S*)-norketamine is a more effective antidepressant than (*R*)-norketamine. (*S*)-norketamine shows potent antidepressant effects in mice, which continue despite the administration of alpha-amino-3-hyroxy-5-methyl-4-isoxazole-propionic acid receptors (AMPAR) antagonists, suggesting that other targets are involved [90,93]. In an inflammatory model of depression, (*S*)-norketamine’s stereospecific efficacy could be related to esketamine’s greater analgesic effects, as stated earlier. Additionally, (*S*)-norketamine inhibits NMDAR with eight-fold more strength than (*R*)-norketamine, continuing the trend of the (*S*)-enantiomer’s selectivity for NMDAR and its subsequent pathways [94]. Following the oral administration of esketamine, sufficient concentrations of (*S*)-norketamine occur to cause analgesic effects despite oral ketamine’s poor availability [94]. However, norketamine induces urothelial cell death through mitochondrial-mediated apoptosis, which is concerning in the context of repeated use [95].

#### 2.2.4. Hydroxynorketamine

Initially considered inactive, hydroxynorketamine’s effects lack the classical anesthetic or analgesic properties of ketamine with a lower affinity for NMDAR than the metabolites before it [58,80]. Despite its lower affinity for NMDAR, (*2R*,*6R*)-HNK administration produces antidepressant effects in rodents [80], which generated enough interest for a phase I trial starting in early 2023 (ID: NCT04711005, [96]). Since therapeutic levels of (*2R*,*6R*)-HNK is not enough to inhibit NMDAR, its ability to enhance glutamatergic excitatory signaling is of great interest [83,84]. One proposed mechanism involves the inhibition of group II metabotropic glutamate receptors subtype 2 (mGlu2), which follows a similar mechanism to the disinhibition hypothesis (see below), inhibiting a glutamate signaling inhibitor [84]. Arketamine and (*2R*,*6R*)-HNK increase the in vitro production of KCNQ2 mRNA (codes for potassium voltage-gated channel subfamily Q member 2) implicated in depression [97], in ventral hippocampus glutaminergic neurons, adding yet another potential mechanism of anti-depressant activity [97,98]. Improvement in the glutamatergic transmission ultimately leads to AMPAR activation, converging with the NMDAR-dependent mechanism of antidepressant action [58,80]. (*2R*,*6R*)-HNK’s antidepressant mechanism is dependent on activity-dependent BDNF release, lending further credence to its alternate pathway to disinhibition [80,99]. However, one subgroup analysis of a clinical trial found an inverse correlation between (*2R*,*6R*)-HNK levels and improvement in depression and suicidal ideation in depressed patients [100]. The authors pose that this inverse correlation could be due to differences in mouse and human metabolism, or that an inverse blood concentration of HNKs is due to the differences in absorption into the brain [100].

### 2.3. Neuroplasticity and Neuroprotection Mediate Ketamine’s Complex Antidepressant Mechanism of Action

In the past two decades, numerous studies show a link between neurotrophic factors and depression and mood disorders [101]. Ketamine’s mechanism of action in the rapid relief of depressive symptoms is involved in the activity of several neurotrophic factors [29]. BDNF plays a major role. Since its purification in 1982 [102], BDNF has been the focus of numerous studies investigating its role in physiologic neuroplasticity and its potential as a pharmacologic target to treat CNS disorders [101]. BDNF is the most highly expressed member of the neurotrophin family of growth factors. Other members of this family include neurotrophin 3 and neurotrophin 4, as well as nerve growth factor (NGF) [103]. Initially, in animal models, convergent increases in BDNF followed both chronic electroconvulsive seizure and antidepressant therapy [104]. Several large studies done with human subjects delineate the utility of BDNF as a possible pharmacologic target or a biomarker for depression. A large meta-analysis (*n* = 748) shows that depressant subjects had significantly lower serum BDNF levels than non-depressed patients, and found that their serum BDNF levels rose following antidepressant therapy [105]. A more recent similar meta-analysis repeated this observation that antidepressant classes can raise serum BDNF levels, though the same effect was only observed with sertraline when applied to individual drugs [106]. Postmortem studies of suicide victims also observe possible differences in BDNF expression within brain tissue when compared to healthy controls. A systematic review of eight studies (*n* = 684) provides data that BDNF dysregulation could play a role in depression, though more research is needed to provide additional evidence that decreased BDNF levels are consistently seen in a completed suicide [107]. In addition to studies involving serum BDNF levels, several studies reveal the connection between a BDNF polymorphism, Val66Met, to depression. Val66Met affects the activity-dependent release of BDNF [108]. A meta-analysis in 2012 (*n* = 523) shows a link between the susceptibility to depression in old age and this polymorphism [109]. Similarly, more recent meta-analyses (*n* = 21,060) show a relationship between stress and depression with the Val66Met BDNF polymorphism [110]. Although these correlations need further investigation, they provide compelling evidence that the monoamine hypothesis may not completely explain the underlying mechanisms of depression and mood disorders. More research is needed to elucidate the pathophysiological link between BDNF dysregulation and depression. However, the available evidence strongly suggests that neurotrophic factors such as BDNF most likely play key roles in the maintenance of mental health.

BDNF is expressed throughout the brain, though it is highly expressed in the hippocampus and the cerebral cortex [103]. BDNF is initially synthesized as pre-pro-BDNF and is further cleaved to pro-BDNF. It is then cleaved into mature BDNF, which has been implicated as the active product [111]. It is not clear whether BDNF is specifically released from presynaptic or postsynaptic sites. Immunofluorescent antibody studies suggest that BDNF is packaged solely into presynaptic vesicles and is involved in synaptic signaling in an anterograde fashion [112]. Another prevailing view is that BDNF is synthesized pre- and post-synaptically, where it is involved in autocrine and paracrine signaling on the pre- and postsynaptic membranes [108]. Regardless, the release of BDNF appears to be dependent on intracellular calcium levels and intracellular calcium influx in normal physiological states likely related to the activity of NMDAR and AMPAR activity [113].

BDNF plays an essential role as a ligand in several key signaling pathways. It has a high affinity for the tropomyosin receptor kinase B (TrkB) [114], which is expressed both in presynaptic axon terminals and postsynaptic dendritic spines [115]. A cAMP/Ca^2+^-dependent mechanism heavily regulates TrkB surface translocation. The activity of the NMDAR and AMPAR likely plays a role in TrkB surface translocation as well [116]. BDNF and TrkB binding activates three primary intracellular signal cascades: the MAPK pathway, the PI3K-Akt pathway, and the PLC-γ pathway. The PI3K-Akt pathway appears to be related to BDNF’s anti-apoptotic and pro-survival effects, while the MAPK and PLC-γ pathway is implicated in BDNF’s modulation of synaptic plasticity [103,108]. Studies in many animal models reveal that BDNF is essential during development. BDNF knockout mice often die before reaching maturity [112]. In the adult human brain, BDNF appears to play a role in long-term potentiation, which is the strengthening of the connection between synapses that is believed to play a vitally important role in memory formation [117,118].

### 2.4. Ketamine and the Disinhibition Hypothesis

The most substantiated mechanism of ketamine’s antidepressant action is the disinhibition hypothesis, which is primarily focused on glutamate neurotransmission [119,120]. This notion posits that ketamine’s action is related to the inhibition of GABAergic interneurons in the prefrontal cortex and hippocampus, which leads to downstream glutamatergic stimulation and synaptic plasticity. Ketamine rapidly increases glutamate levels in the prefrontal cortex and hippocampus using subanesthetic doses in rats. These subanesthetic doses increase levels of glutamate, while higher anesthetic doses diminish the release of glutamate [121]. Ketamine also increases glutamate cycling in the PFC [122] and directly increases glutamate neurotransmission in the mPFC and hippocampus. The inhibition of GABAergic interneurons within the hippocampus accompanies this increase in glutamate [123,124]. Glutamate release from the CA1 (cornu ammonis 1) region of the hippocampus has a direct, dose-dependent effect on the expression and release of BDNF, as well as the expression and translocation of TrkB receptors in this brain region. This provides evidence for the causal link between increased synaptic glutamate mediating the release of BDNF, supporting the disinhibition hypothesis [125]. The spike in glutamate levels within the mPFC and hippocampus is then implicated in the activation of post-synaptic AMPAR. AMPARs are ionotropic glutamate receptors with a specific function in rapid neurotransmission. Importantly, they are also implicated in synaptic plasticity [126,127]. The inhibition of the glutamine reuptake receptor, GLT-1, in glial cells produces a similar antidepressant effect to ketamine in mouse models [128]. However, a separate study showed that GLT-1 inhibition blocked the antidepressant-like effects of ketamine and altered downstream phosphorylation [129]. This effect was attributed to the deregulation of glutamate cycling leading to overstimulation and excitotoxicity.

The downstream effects of AMPAR stimulation involve the release of BDNF, which interacts with post-synaptic TrkB receptors. Ketamine increases AMPAR synaptic transmission, specifically in the hippocampus [130]. Several studies show that AMPAR activation is necessary for the antidepressant actions of ketamine. In mouse models of depression, AMPAR inhibition had either diminished or fully eradicated the antidepressant effects of ketamine [131,132,133,134]. Furthermore, pre-treatment with AMPAR antagonists decreases levels of BDNF and the mechanistic target of rapamycin (mTOR) (a downstream effector of BDNF-TrkB signaling discussed below) in the prefrontal cortex and hippocampus. Pre-treatment with AMPAR antagonists also diminishes the antidepressant actions of ketamine, while pretreatment with an AMPAR agonist increases levels of BDNF and mTOR and augments ketamine’s antidepressant effects [135]. Recent evidence now demonstrates that even TrkB receptor antagonists can block the therapeutic effects of ketamine [136]. Taken altogether, this data suggests that the AMPA-mediated release of BDNF and BDNF’s action on TrkB receptor-mediated cell signaling play a cardinal role in the rapid antidepressant effects of ketamine.

**Figure 2:**
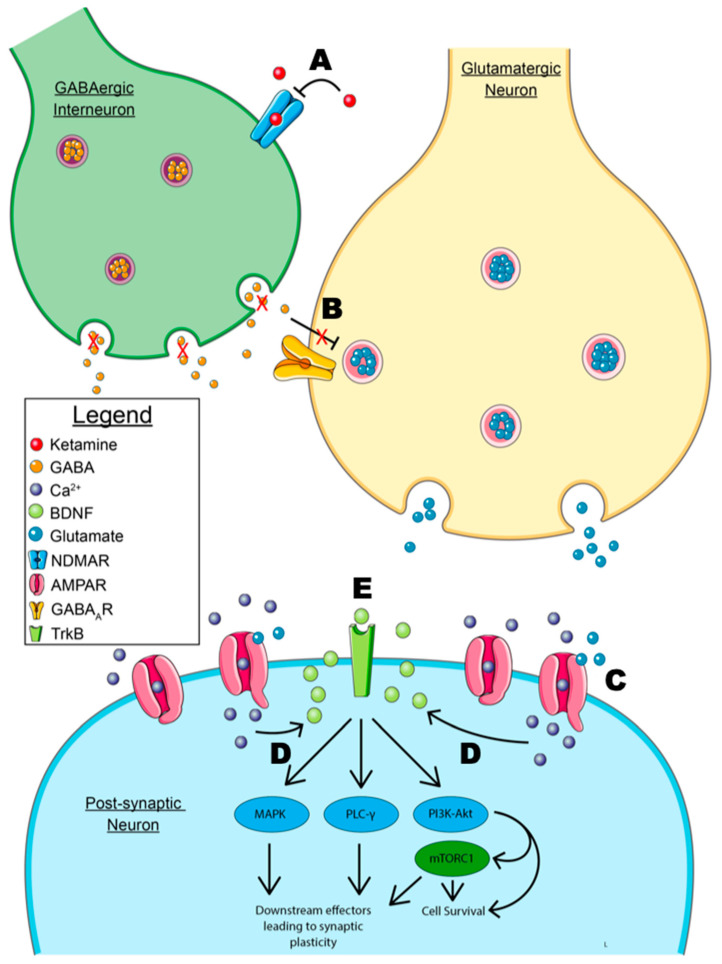
**Model of the disinhibition hypothesis. A.** Ketamine’s antidepressant mechanism of action primarily depends on the antagonism of NMDARs (N-methyl-D-aspartate receptors) on GABAergic interneurons preventing GABA release. **B.** The inhibition of GABA release prevents the inhibition of pyramidal glutamatergic neurons. This allows for the release of glutamate and the downstream effects of the subsequent glutamate surge. **C.** Glutamate binds to post-synaptic AMPARs (α-amino-3-hydroxy-5-methyl-4-isoxazolepropionic acid receptor), allowing for calcium influx. **D.** Calcium influx leads to the calcium-dependent release of BDNF from the post-synaptic membrane. NOTE: the model shows the post-synaptic release of BDNF, though an immunofluorescent localization study has suggested that the pre-synaptic release of BDNF at the downstream synaptic cleft is involved [112]. **E.** Autocrine signaling of BDNF leads to downstream signaling through the MAPK, PLC-γ, and PI3K-Akt signaling pathways. The MAPK and PLC-γ pathway are primarily implicated in synaptic plasticity, while the PI3k-Akt pathway leads to anti-apoptotic signaling and cell survival. Signaling through mTORC1 has also been implicated in synaptic plasticity and neuritogenesis. NOTE: As mentioned previously, autocrine signaling is shown, but paracrine signaling may be involved based on the location of BNDF-containing vesicles in immunofluorescence studies [112]. Some graphic components from Servier Medical Art were used to draw parts of this model. Servier Medical Art by Servier is licensed under a Creative Commons Attribution 3.0 Unported License (https://creativecommons.org/licenses/by/3.0/, accessed 3 May 2023).

The elucidation of downstream effectors that play a role in ketamine’s mechanism of action has provided a difficult challenge. As previously mentioned, BDNF has several downstream effectors that are implicated in synaptic plasticity. One specific effector of interest involves downstream actions of the phosphatidylinositol-3-kinase (PI3K)-Akt and the mitogen-activated protein kinase kinase-extracellular signal-regulated kinase (MEK-ERK) pathways leading to the activation of mTOR, specifically mTORC1 [137]. mTOR is ubiquitously expressed and has numerous functions in cell proliferation, survival, and growth [138]. These pathways, as previously stated, are implicated in the survival of neurons, neuritogenesis, and synaptic plasticity [26]. Additionally, dysregulation of the MEK-ERK pathway is implicated in depression [139]. Recent data show that mTOR knockdowns in the infralimbic cortex induced a depressive state in mice [140]. Mouse models also reveal that mTOR inhibition with rapamycin directly infused via intracerebroventricular injection completely abolishes the antidepressant actions of ketamine [27,141]. However, rapamycin administration to human patients undergoing ketamine therapy greatly increases the duration of ketamine’s antidepressant effects [142]. While this might suggest that ketamine’s mechanism of action in the treatment of depression is independent of mTOR activation, it is likely that the route of administration (oral formulation of rapamycin) and peripheral decrease of inflammation is the reason for these findings. Interestingly, rapamycin pretreatment has no effect on suicidal ideation during a depressive intervention with ketamine, suggesting that mTOR may not mediate this comorbidity [143]. The major events of the disinhibition hypothesis for ketamine’s mechanism of action are depicted in Figure 2.

### 2.5. Other Potential Growth Factors Involved in Ketamine’s Action

#### 2.5.1. VEGF

VEGF may play a role in MDD [144] and chronic stress [145]. VEGF stimulates neurogenesis both in vitro and in vivo [146]. The activation of TrkB increases VEGF levels in neuroblastoma cells and could play a direct role in tumor progression. Thus, a question arises as to whether VEGF plays a role in BDNF-related neurogenesis or even neuritogenesis, and if VEGF plays a role in ketamine’s mechanism of action. VEGF expression in the basal hippocampus was partially necessary for the antidepressant effects of ketamine [147]. More recently, the co-infusion of BDNF and VEGF-neutralizing antibodies into rat brains block the antidepressant actions of direct infusions of BDNF [148]. Furthermore, deletion of the VEGF gene, the VEGF receptor, Flk-1, or infusions of VEGF neutralizing antibodies into the mPFC blocks the antidepressant actions of ketamine in mice [149]. To address the role of VEGF in humans, recent studies have examined the plasma level of VEGF during the treatment response with ketamine. Plasma VEGF is not associated with the antianhedonic effects of intravenous ketamine [150]. Additionally, plasma VEGF levels are not related to ketamine’s effects on suicidal ideation [151]. Finally, plasma VEGF does not predict ketamine treatment response in depressed patients using a MADRS rating scale [150]. These observations suggest that plasma VEGF plays no role in the antidepressant actions of ketamine. However, a simple view of these results would be that although serum VEGF levels in patients do not predict treatment response, they could very well be responsible for its actions in the brain when taken with previous animal models. Testing that idea is difficult with our current technology.

#### 2.5.2. IGF-1

Insulin-like growth factor 1 (IGF-1) is a polypeptide hormone that plays a pivotal role in the development of the brain, and recent evidence suggests that it may play a role in the pathogenesis of depression. It is widely expressed throughout the body, and during embryonic development in mice it is highly expressed in nervous tissues, particularly in the hippocampus, olfactory bulbs, and cerebellum [151]. Mice lineages that overexpress IGF-1 show drastic increases in brain size that are directly due to increased numbers of neurons [152,153], while mice that under-express IGF-1 show profound developmental delays and abnormalities of several brain regions [154,155]. A potential link between decreases in IGF-1 expression and depression in rat models [156] and peripheral IGF-1 levels could potentially be a marker of depression in human subjects with MDD and bipolar disorder [157]. In addition to the possible role of IGF-1 dysregulation in depression, IGF-1 modulation within the prefrontal cortex plays a role in the mechanism of action of fluoxetine [158]. Furthermore, IGF-1 infusions systemically, centrally, and into the mPFC can also elicit an antidepressant effect [159,160,161].

A recent study shows that ketamine significantly increases IGF-1 levels in the mPFC. To test the potential role of this IGF-1 modulation in ketamine’s antidepressant effects, anti-IGF-1 antibodies were administered either 15 min before or 2 h after ketamine infusion. The antibody pre and post-treatment blocked the antidepressant effects of ketamine, as measured in multiple experimental models of depression regardless of the sequence of intervention (antibodies first or ketamine infusion first) [162]. This implicates IGF-1 as a requirement for the antidepressant action of ketamine. Furthermore, IGF-I modulates BDNF expression [163], and this could potentially indicate that IGF-1 plays an essential role in glutamate-mediated BDNF release. However, more investigation into the role of IGF-1 in ketamine’s antidepressant effect is needed to further delineate this possible connection.

## 3. Conclusions

Ketamine’s pharmacological and rapid antidepressant properties make it of keen interest in clinical settings, especially in treating TRD. Ketamine stimulates glutamate surge, BDNF release, and downstream effects on TrkB, giving it potent activity on synaptic plasticity, synaptogenesis, and modulating neuroprotection. More research is needed to reveal the specific roles of VEGF and IGF-1. Additionally, intranasal agents such as arketamine and esketamine are of particular interest, though arketamine appears to have superior antidepressant efficacy, a longer effect time, and minimal side effects compared to esketamine. One sub-anesthetic dose of ketamine is also a promising, rapid treatment for suicidal ideation due to ketamine’s prompt cascade of effects within hours of administration.

Current antidepressant interventions require long-term consistent use and have significant latency periods between the initiation of therapy and the desired therapeutic outcomes. Ketamine’s rapid effects and limited side effects can increase patient compliance, as fewer doses equate to the effects of weeks to months of traditional pharmacological treatments. One specific shortcoming of ketamine infusions is its rapidly dissipating effects, with many clinical trials showing its effectiveness beginning to wane only after 2–4 weeks post-infusion [164]. Drug-assisted psychotherapy with novel rapid-acting psychoactive agents such as MDMA is highly successful in the treatment of other psychiatric conditions such as PTSD [165], with effects sustained up to 12 months post-treatment [166]. A similar ketamine-assisted psychotherapy paradigm could potentially increase the duration of ketamine treatment and further reduce the number of treatments required to sustain the antidepressant effects for prolonged periods.

Combining psychotherapy with medications may lead to superior therapeutic outcomes. SSRIs and SNRIs are commonly coupled with “talk therapy” to maximize benefits. More research is needed on the use of esketamine when combined with psychotherapy. This biopsychosocial perspective addresses both the intrinsic and extrinsic factors of MDD and TRD. In addition to MDD and TRD, ketamine’s promising effects on bipolar disorder [43,44], postpartum depression [167,168,169], Alzheimer’s disease [170], suicidality [171], and remission from other psychiatric disorders such as PTSD [165,166,172] are still being investigated.

## Figures and Tables

**Table 1:** A Repertoire of Ketamine Targets (derived from [53]).

Target	Action	Comments	Reference
Glutamate receptor ionotropic, NMDA 3A	antagonist	blocks the open ion channel directly and through negative allosteric regulation; prevents the activation of a calcium-dependent NO synthetase, which plays a role in nociception and neurotoxicity	[59]
5-hydroxytryptamine receptor 3A	potentiator	increases voltage-gated potassium channel activity; binds at supratherapeutic doses, and is thought to increase the effects of the receptor through indirect mechanisms	[60]
α-7 nicotinic cholinergic receptor subunit	antagonist	its effects on skeletal muscle tone are not noticed unless unmasked by additional muscle relaxants; ketamine’s NMDAR antagonism additionally inhibits acetylcholine release through the receptors	[61]
Muscarinic acetylcholine receptor M1	inhibitor	primarily found in the hippocampus and the cerebral cortex	[62]
Nitric oxide synthase	indirect inhibitor	in the brain; functions through the glutamate/NO/cGMP system; may contribute to neuroprotective, sympathetic activating, and additional analgesic effects	[63]
Neurokinin 1 receptor	antagonist	through noncompetitive inhibition; possibly contributes to an analgesic effect, as this receptor modulates spinal cord nociception, but the therapeutic relevance of this interaction is not fully clear	[64]
Dopamine D2 receptor	agonist/partial agonist	specifically binds to the high-affinity state of the receptor; binding is more than 10 times weaker than that of dopamine and phencyclidine	[65]
Opioid receptors	mild agonist	binding affinity from strongest to weakest: mu > kappa > delta; related to some analgesic properties and adverse side effects, particularly with the kappa receptor	[66,67]
Sodium-dependent noradrenaline transporter	inhibitor	blocks reuptake in the heart, leading to increased chronotropy and vasoconstriction	[68]

## Data Availability

No new data were created during this work.

## References

[1] World Health Organization Depression. https://www.who.int/news-room/fact-sheets/detail/depression.

[2] National Institute of Mental Health Major Depression. https://www.nimh.nih.gov/health/statistics/major-depression.

[3] American Psychiatric Association (2022). Diagnostic and Statistical Manual of Mental Disorders.

[4] Angst J., Angst F., Stassen H.H. (1999). Suicide Risk in Patients with Major Depressive Disorder. J. Clin. Psychiatry.

[5] Karrouri R., Hammani Z., Benjelloun R., Otheman Y. (2021). Major Depressive Disorder: Validated Treatments and Future Challenges. World J. Clin. Cases.

[6] Clevenger S.S., Malhotra D., Dang J., Vanle B., IsHak W.W. (2018). The Role of Selective Serotonin Reuptake Inhibitors in Preventing Relapse of Major Depressive Disorder. Ther. Adv. Psychopharmacol..

[7] Kirwin J.L., Gören J.L. (2005). Duloxetine: A Dual Serotonin-Norepinephrine Reuptake Inhibitor for Treatment of Major Depressive Disorder. Pharmacotherapy.

[8] Sverre K.T., Nissen E.R., Farver-Vestergaard I., Johannsen M., Zachariae R. (2023). Comparing the Efficacy of Mindfulness-Based Therapy and Cognitive-Behavioral Therapy for Depression in Head-to-Head Randomized Controlled Trials: A Systematic Review and Meta-Analysis of Equivalence. Clin. Psychol. Rev..

[9] Bassa A., Sagués T., Porta-Casteràs D., Serra P., Martínez-Amorós E., Palao D., Cano M., Cardoner N. (2021). The Neurobiological Basis of Cognitive Side Effects of Electroconvulsive Therapy: A Systematic Review. Brain Sci..

[10] Cipriani A., Furukawa T.A., Salanti G., Chaimani A., Atkinson L.Z., Ogawa Y., Leucht S., Ruhe H.G., Turner E.H., Higgins J.P.T. (2018). Comparative Efficacy and Acceptability of 21 Antidepressant Drugs for the Acute Treatment of Adults with Major Depressive Disorder: A Systematic Review and Network Meta-Analysis. Lancet.

[11] Voineskos D., Daskalakis Z.J., Blumberger D.M. (2020). Management of Treatment-Resistant Depression: Challenges and Strategies. Neuropsychiatr. Dis. Treat..

[12] Machado-Vieira R., Baumann J., Wheeler-Castillo C., Latov D., Henter I., Salvadore G., Zarate C. (2010). The Timing of Antidepressant Effects: A Comparison of Diverse Pharmacological and Somatic Treatments. Pharmaceuticals.

[13] Kennedy S.H., Lam R.W., McIntyre R.S., Tourjman S.V., Bhat V., Blier P., Hasnain M., Jollant F., Levitt A.J., MacQueen G.M. (2016). Canadian Network for Mood and Anxiety Treatments (CANMAT) 2016 Clinical Guidelines for the Management of Adults with Major Depressive Disorder. Can. J. Psychiatry.

[14] Pandarakalam J.P. (2018). Challenges of Treatment-Resistant Depression. Psychiatr. Danub..

[15] Kverno K.S., Mangano E. (2021). Treatment-Resistant Depression: Approaches to Treatment. J. Psychosoc. Nurs. Ment. Health Serv..

[16] Zhdanava M., Pilon D., Ghelerter I., Chow W., Joshi K., Lefebvre P., Sheehan J.J. (2021). The Prevalence and National Burden of Treatment-Resistant Depression and Major Depressive Disorder in the United States. J. Clin. Psychiatry.

[17] Kendler K.S. (2020). The Origin of Our Modern Concept of Depression-The History of Melancholia from 1780–1880: A Review. JAMA Psychiatry.

[18] Moncrieff J., Cooper R.E., Stockmann T., Amendola S., Hengartner M.P., Horowitz M.A. (2022). The Serotonin Theory of Depression: A Systematic Umbrella Review of the Evidence. Mol. Psychiatry.

[19] Ayala-Lopez N., Watts S.W. (2021). Physiology and Pharmacology of Neurotransmitter Transporters. Comprehensive Physiology.

[20] Sansone R.A., Sansone L.A. (2014). Serotonin Norepinephrine Reuptake Inhibitors: A Pharmacological Comparison. Innov. Clin. Neurosci..

[21] Xue W., Wang P., Li B., Li Y., Xu X., Yang F., Yao X., Chen Y.Z., Xu F., Zhu F. (2016). Identification of the Inhibitory Mechanism of FDA Approved Selective Serotonin Reuptake Inhibitors: An Insight from Molecular Dynamics Simulation Study. Phys. Chem. Chem. Phys..

[22] Nakamura S. (2023). Perspective Chapter: Depression as a Disorder of Monoamine Axon Degeneration May Hold an Answer to Two Antidepressant Questions-Delayed Clinical Efficacy and Treatment-Resistant Depression. COVID-19 Pandemic, Mental Health and Neuroscience-New Scenarios for Understanding and Treatment.

[23] Mosiołek A., Mosiołek J., Jakima S., Pięta A., Szulc A. (2021). Effects of Antidepressant Treatment on Neurotrophic Factors (BDNF and IGF-1) in Patients with Major Depressive Disorder (MDD). J. Clin. Med..

[24] Price R.B., Duman R. (2020). Neuroplasticity in Cognitive and Psychological Mechanisms of Depression: An Integrative Model. Mol. Psychiatry.

[25] Duman R.S. (2014). Pathophysiology of Depression and Innovative Treatments: Remodeling Glutamatergic Synaptic Connections. Dialogues Clin. Neurosci..

[26] Duman R.S., Aghajanian G.K., Sanacora G., Krystal J.H. (2016). Synaptic Plasticity and Depression: New Insights from Stress and Rapid-Acting Antidepressants. Nat. Med..

[27] Li N., Liu R.-J., Dwyer J.M., Banasr M., Lee B., Son H., Li X.-Y., Aghajanian G., Duman R.S. (2011). Glutamate N-Methyl-D-Aspartate Receptor Antagonists Rapidly Reverse Behavioral and Synaptic Deficits Caused by Chronic Stress Exposure. Biol. Psychiatry.

[28] Dogra S., Conn P.J. (2021). Targeting Metabotropic Glutamate Receptors for the Treatment of Depression and Other Stress-Related Disorders. Neuropharmacology.

[29] Deyama S., Kaneda K. (2023). Role of Neurotrophic and Growth Factors in the Rapid and Sustained Antidepressant Actions of Ketamine. Neuropharmacology.

[30] Ly C., Greb A.C., Vargas M.V., Duim W.C., Grodzki A.C.G., Lein P.J., Olson D.E. (2021). Transient Stimulation with Psychoplastogens Is Sufficient to Initiate Neuronal Growth. ACS Pharmacol. Transl. Sci..

[31] Domino E.F., Warner D.S. (2010). Taming the Ketamine Tiger. Anesthesiology.

[32] Cohen B.D. (1962). Comparison of Phencyclidine Hydrochloride (Sernyl) with Other Drugs. Arch. Gen. Psychiatry.

[33] Sleigh J., Harvey M., Voss L., Denny B. (2014). Ketamine–More Mechanisms of Action than Just NMDA Blockade. Trends Anaesth. Crit. Care.

[34] Mion G. (2017). History of Anaesthesia -The Ketamine Story–Past, Present and Future. Eur. J. Anaesthesiol.

[35] Hashimoto K. (2019). Rapid-acting Antidepressant Ketamine, Its Metabolites and Other Candidates: A Historical Overview and Future Perspective. Psychiatry Clin. Neurosci..

[36] Tyler M.W., Yourish H.B., Ionescu D.F., Haggarty S.J. (2017). Classics in Chemical Neuroscience: Ketamine. ACS Chem. Neurosci..

[37] Berman R.M., Cappiello A., Anand A., Oren D.A., Heninger G.R., Charney D.S., Krystal J.H. (2000). Antidepressant Effects of Ketamine in Depressed Patients. Biol. Psychiatry.

[38] Zarate C.A., Singh J.B., Carlson P.J., Brutsche N.E., Ameli R., Luckenbaugh D.A., Charney D.S., Manji H.K. (2006). A Randomized Trial of an N-Methyl-D-Aspartate Antagonist in Treatment-Resistant Major Depression. Arch. Gen. Psychiatry.

[39] Su T.-P., Chen M.-H., Li C.-T., Lin W.-C., Hong C.-J., Gueorguieva R., Tu P.-C., Bai Y.-M., Cheng C.-M., Krystal J.H. (2017). Dose-Related Effects of Adjunctive Ketamine in Taiwanese Patients with Treatment-Resistant Depression. Neuropsychopharmacology.

[40] Price R.B., Nock M.K., Charney D.S., Mathew S.J. (2009). Effects of Intravenous Ketamine on Explicit and Implicit Measures of Suicidality in Treatment-Resistant Depression. Biol. Psychiatry.

[41] Larkin G.L., Beautrais A.L. (2011). A Preliminary Naturalistic Study of Low-Dose Ketamine for Depression and Suicide Ideation in the Emergency Department. Int. J. Neuropsychopharmacol..

[42] FDA FDA Approves New Nasal Spray Medication for Treatment-Resistant Depression; Available Only at a Certified Doctor’s Office or Clinic. https://www.fda.gov/news-events/press-announcements/fda-approves-new-nasal-spray-medication-treatment-resistant-depression-available-only-certified.

[43] Zheng W., Zhou Y.-L., Liu W.-J., Wang C.-Y., Zhan Y.-N., Li H.-Q., Chen L.-J., Li M.D., Ning Y.-P. (2018). Rapid and Longer-Term Antidepressant Effects of Repeated-Dose Intravenous Ketamine for Patients with Unipolar and Bipolar Depression. J. Psychiatr. Res..

[44] Zarate C.A., Brutsche N.E., Ibrahim L., Franco-Chaves J., Diazgranados N., Cravchik A., Selter J., Marquardt C.A., Liberty V., Luckenbaugh D.A. (2012). Replication of Ketamine’s Antidepressant Efficacy in Bipolar Depression: A Randomized Controlled Add-on Trial. Biol. Psychiatry.

[45] d’Andrea G., Pettorruso M., Di Lorenzo G., Mancusi G., McIntyre R.S., Martinotti G. (2023). Rethinking Ketamine and Esketamine Action: Are They Antidepressants with Mood-Stabilizing Properties?. Eur. Neuropsychopharmacol..

[46] Diazgranados N., Ibrahim L., Brutsche N.E., Newberg A., Kronstein P., Khalife S., Kammerer W.A., Quezado Z., Luckenbaugh D.A., Salvadore G. (2010). A Randomized Add-on Trial of an N-Methyl-D-Aspartate Antagonist in Treatment-Resistant Bipolar Depression. Arch. Gen. Psychiatry.

[47] Lee Y., Syeda K., Maruschak N.A., Cha D.S., Mansur R.B., Wium-Andersen I.K., Woldeyohannes H.O., Rosenblat J.D., McIntyre R.S. (2016). A New Perspective on the Anti-Suicide Effects With Ketamine Treatment: A Procognitive Effect. J. Clin. Psychopharmacol..

[48] Duman R.S., Deyama S., Fogaça M.V. (2021). Role of BDNF in the Pathophysiology and Treatment of Depression: Activity-dependent Effects Distinguish Rapid-acting Antidepressants. Eur. J. Neurosci..

[49] Aleksandrova L.R., Wang Y.T., Phillips A.G. (2020). Ketamine and Its Metabolite, (2R,6R)-HNK, Restore Hippocampal LTP and Long-Term Spatial Memory in the Wistar-Kyoto Rat Model of Depression. Mol. Brain.

[50] National Center for Biotechnology Information PubChem Compound Summary for CID 3821, Ketamine. https://pubchem.ncbi.nlm.nih.gov/compound/Ketamine.

[51] Djoumbou Feunang Y., Eisner R., Knox C., Chepelev L., Hastings J., Owen G., Fahy E., Steinbeck C., Subramanian S., Bolton E. (2016). ClassyFire: Automated Chemical Classification with a Comprehensive, Computable Taxonomy. J. Cheminform..

[52] Hastings J., Owen G., Dekker A., Ennis M., Kale N., Muthukrishnan V., Turner S., Swainston N., Mendes P., Steinbeck C. (2016). ChEBI in 2016: Improved Services and an Expanding Collection of Metabolites. Nucleic Acids Res..

[53] Wishart D.S., Feunang Y.D., Guo A.C., Lo E.J., Marcu A., Grant J.R., Sajed T., Johnson D., Li C., Sayeeda Z. (2018). DrugBank 5.0: A Major Update to the DrugBank Database for 2018. Nucleic Acids Res..

[54] Botanas C.J., de la Peña J.B., Kim H.J., Lee Y.S., Cheong J.H. (2019). Methoxetamine: A Foe or Friend?. Neurochem. Int..

[55] Wishart D.S., Guo A., Oler E., Wang F., Anjum A., Peters H., Dizon R., Sayeeda Z., Tian S., Lee B.L. (2022). HMDB 5.0: The Human Metabolome Database for 2022. Nucleic Acids Res..

[56] Anis N.A., Berry S.C., Burton N.R., Lodge D. (1983). The Dissociative Anaesthetics, Ketamine and Phencyclidine, Selectively Reduce Excitation of Central Mammalian Neurones by N-Methyl-Aspartate. Br. J. Pharmacol..

[57] Thomson A.M., West D.C., Lodge D. (1985). An N-Methylaspartate Receptor-Mediated Synapse in Rat Cerebral Cortex: A Site of Action of Ketamine?. Nature.

[58] Zanos P., Moaddel R., Morris P.J., Georgiou P., Fischell J., Elmer G.I., Alkondon M., Yuan P., Pribut H.J., Singh N.S. (2016). NMDAR Inhibition-Independent Antidepressant Actions of Ketamine Metabolites. Nature.

[59] Sinner B., Graf B.M., Schüttler J., Schwilden H. (2008). Ketamine. Modern Anesthetics.

[60] Appadu B.L., Lambert D.G. (1996). Interaction of i.v. Anaesthetic Agents with 5-HT3 Receptors. Br. J. Anaesth..

[61] Ho K.K., Flood P. (2004). Single Amino Acid Residue in the Extracellular Portion of Transmembrane Segment 2 in the Nicotinic A7 Acetylcholine Receptor Modulates Sensitivity to Ketamine. Anesthesiology.

[62] Durieux M.E. (1995). Inhibition by Ketamine of Muscarinic Acetylcholine Receptor Function. Anesth. Analg..

[63] Mahmoudzade S., Goudarzi S., Mohammad Jafari R., Shafaroodi H., Dehpour A.R., Sanatkar M. (2022). The N-methyl-D-aspartate Receptor Antagonist Ketamin Exerts Analgesic Effects via Modulation of the Nitric Oxide Pathway. Fundam. Clin. Pharmacol..

[64] Soares P.C.L.R., Corrêa J.M.X., Niella R.V., de Oliveira J.N.S., Costa B.A., Silva Junior A.C., Sena A.S., Pinto T.M., Munhoz A.D., Martins L.A.F. (2021). Continuous Infusion of Ketamine and Lidocaine Either with or without Maropitant as an Adjuvant Agent for Analgesia in Female Dogs Undergoing Mastectomy. Vet. Med. Int..

[65] Seeman P., Guan H.-C., Hirbec H. (2009). Dopamine D2 High Receptors Stimulated by Phencyclidines, Lysergic Acid Diethylamide, Salvinorin A, and Modafinil. Synapse.

[66] Hustveit O., Maurset A., Øye I. (1995). Interaction of the Chiral Forms of Ketamine with Opioid, Phencyclidine, σ and Muscarinic Receptors. Pharmacol. Toxicol..

[67] Kohrs R., Durieux M.E. (1998). Ketamine-Teaching an Old Drug New Tricks. Anesth. Analg..

[68] Salt P.J., Barnes P.K., Beswick F.J. (1979). Inhibition of Neuronal and Extraneuronal Uptake of Noradrenaline by Ketamine in the Isolated Perfused Rat Heart. Br. J. Anaesth..

[69] Vadivelu N., Schermer E., Kodumudi V., Belani K., Urman R., Kaye A. (2016). Role of Ketamine for Analgesia in Adults and Children. J. Anaesthesiol. Clin. Pharmacol..

[70] Li L., Vlisides P.E. (2016). Ketamine: 50 Years of Modulating the Mind. Front Hum Neurosci..

[71] Kharasch E.D., Labroo R. (1992). Metabolism of Ketamine Stereoisomers by Human Liver Microsomes. Anesthesiology.

[72] Wei Y., Chang L., Hashimoto K. (2022). Molecular Mechanisms Underlying the Antidepressant Actions of Arketamine: Beyond the NMDA Receptor. Mol. Psychiatry.

[73] Yang C., Yang J., Luo A., Hashimoto K. (2019). Molecular and Cellular Mechanisms Underlying the Antidepressant Effects of Ketamine Enantiomers and Its Metabolites. Transl. Psychiatry.

[74] Clements J.A., Nimmo W.S., Grant I.S. (1982). Bioavailability, Pharmacokinetics, and Analgesic Activity of Ketamine in Humans. J. Pharm. Sci..

[75] McIntyre R.S., Rosenblat J.D., Nemeroff C.B., Sanacora G., Murrough J.W., Berk M., Brietzke E., Dodd S., Gorwood P., Ho R. (2021). Synthesizing the Evidence for Ketamine and Esketamine in Treatment-Resistant Depression: An International Expert Opinion on the. Available Evidence and Implementation. Am. J. Psychiatry.

[76] Ihmsen H. (2001). Stereoselective Pharmacokinetics of Ketamine: R(–)-Ketamine Inhibits the Elimination of S(+)-Ketamine. Clin. Pharmacol. Ther..

[77] Kaka J.S., Hayton W.L. (1980). Pharmacokinetics of Ketamine and Two Metabolites in the Dog. J. Pharm. Biopharm..

[78] Hess E.M., Riggs L.M., Michaelides M., Gould T.D. (2022). Mechanisms of Ketamine and Its Metabolites as Antidepressants. Biochem. Pharmacol..

[79] Fanta S., Kinnunen M., Backman J.T., Kalso E. (2015). Population Pharmacokinetics of S-Ketamine and Norketamine in Healthy Volunteers after Intravenous and Oral Dosing. Eur. J. Clin. Pharmacol..

[80] Highland J.N., Zanos P., Riggs L.M., Georgiou P., Clark S.M., Morris P.J., Moaddel R., Thomas C.J., Zarate C.A., Pereira E.F.R. (2021). Hydroxynorketamines: Pharmacology and Potential Therapeutic Applications. Pharmacol. Rev..

[81] Weiss M., Siegmund W. (2022). Pharmacokinetic Modeling of Ketamine Enantiomers and Their Metabolites After Administration of Prolonged-Release Ketamine with Emphasis on 2,6-Hydroxynorketamines. Clin. Pharmacol. Drug Dev..

[82] Highland J.N., Morris P.J., Konrath K.M., Riggs L.M., Hagen N.R., Zanos P., Powels C.F., Moaddel R., Thomas C.J., Wang A.Q. (2022). Hydroxynorketamine Pharmacokinetics and Antidepressant Behavioral Effects of (2,6)- and (5R)-Methyl-(2R,6R)-Hydroxynorketamines. ACS Chem. Neurosci..

[83] Lumsden E.W., Troppoli T.A., Myers S.J., Zanos P., Aracava Y., Kehr J., Lovett J., Kim S., Wang F.-H., Schmidt S. (2019). Antidepressant-Relevant Concentrations of the Ketamine Metabolite (2*R*,6*R*)-Hydroxynorketamine Do Not Block NMDA Receptor Function. Proc. Natl. Acad. Sci. USA.

[84] Zanos P., Highland J.N., Stewart B.W., Georgiou P., Jenne C.E., Lovett J., Morris P.J., Thomas C.J., Moaddel R., Zarate C.A. (2019). (*2R,6R*)-Hydroxynorketamine Exerts MGlu _2_ Receptor-Dependent Antidepressant Actions. Proc. Natl. Acad. Sci. USA.

[85] Malhotra M.D.A. (1997). Ketamine-Induced Exacerbation of Psychotic Symptoms and Cognitive Impairment in Neuroleptic-Free Schizophrenics. Neuropsychopharmacology.

[86] Short B., Fong J., Galvez V., Shelker W., Loo C.K. (2018). Side-Effects Associated with Ketamine Use in Depression: A Systematic Review. Lancet Psychiatry.

[87] Morrison R.L., Fedgchin M., Singh J., Van Gerven J., Zuiker R., Lim K.S., van der Ark P., Wajs E., Xi L., Zannikos P. (2018). Effect of Intranasal Esketamine on Cognitive Functioning in Healthy Participants: A Randomized, Double-Blind, Placebo-Controlled Study. Psychopharmacology.

[88] Wajs E., Aluisio L., Holder R., Daly E.J., Lane R., Lim P., George J.E., Morrison R.L., Sanacora G., Young A.H. (2020). Esketamine Nasal Spray Plus Oral Antidepressant in Patients With Treatment-Resistant Depression. J. Clin. Psychiatry.

[89] Leal G.C., Bandeira I.D., Correia-Melo F.S., Telles M., Mello R.P., Vieira F., Lima C.S., Jesus-Nunes A.P., Guerreiro-Costa L.N.F., Marback R.F. (2021). Intravenous Arketamine for Treatment-Resistant Depression: Open-Label Pilot Study. Eur. Arch. Psychiatry Clin. Neurosci..

[90] Zhang J., Yao W., Hashimoto K. (2022). Arketamine, a New Rapid-Acting Antidepressant: A Historical Review and Future Directions. Neuropharmacology.

[91] Popova V., Daly E.J., Trivedi M., Cooper K., Lane R., Lim P., Mazzucco C., Hough D., Thase M.E., Shelton R.C. (2019). Efficacy and Safety of Flexibly Dosed Esketamine Nasal Spray Combined with a Newly Initiated Oral Antidepressant in Treatment-Resistant Depression: A Randomized Double-Blind Active-Controlled Study. Am. J. Psychiatry.

[92] Monteggia L.M., Gideons E., Kavalali E.T. (2013). The Role of Eukaryotic Elongation Factor 2 Kinase in Rapid Antidepressant Action of Ketamine. Biol. Psychiatry.

[93] Yang C., Kobayashi S., Nakao K., Dong C., Han M., Qu Y., Ren Q., Zhang J., Ma M., Toki H. (2018). AMPA Receptor Activation–Independent Antidepressant Actions of Ketamine Metabolite (S)-Norketamine. Biol. Psychiatry.

[94] Ebert B., Mikkelsen S., Thorkildsen C., Borgbjerg F.M. (1997). Norketamine, the Main Metabolite of Ketamine, Is a Non-Competitive NMDA Receptor Antagonist in the Rat Cortex and Spinal Cord. Eur. J. Pharmacol..

[95] Lin J.-W., Lin Y.-C., Liu J.-M., Liu S.-H., Fang K.-M., Hsu R.-J., Huang C.-F., Chang K.-Y., Lee K.-I., Chang K.-C. (2022). Norketamine, the Main Metabolite of Ketamine, Induces Mitochondria-Dependent and ER Stress-Triggered Apoptotic Death in Urothelial Cells via a Ca^2+^-Regulated ERK1/2-Activating Pathway. Int. J. Mol. Sci..

[96] Raja S., Mack M. ClinicalTrials.gov. https://clinicaltrials.gov/ct2/show/NCT04711005.

[97] Ma L., Hashimoto K. (2022). The Role of Hippocampal KCNQ2 Channel in Antidepressant Actions of Ketamine. Neuron.

[98] Lopez J.P., Lücken M.D., Brivio E., Karamihalev S., Kos A., De Donno C., Benjamin A., Yang H., Dick A.L.W., Stoffel R. (2022). Ketamine Exerts Its Sustained Antidepressant Effects via Cell-Type-Specific Regulation of Kcnq2. Neuron.

[99] Fukumoto K., Fogaça M.V., Liu R.-J., Duman C., Kato T., Li X.-Y., Duman R.S. (2019). Activity-Dependent Brain-Derived Neurotrophic Factor Signaling Is Required for the Antidepressant Actions of (2*R*,6*R*)-Hydroxynorketamine. Proc. Natl. Acad. Sci. USA.

[100] Grunebaum M.F., Galfalvy H.C., Choo T.-H., Parris M.S., Burke A.K., Suckow R.F., Cooper T.B., Mann J.J. (2019). Ketamine Metabolite Pilot Study in a Suicidal Depression Trial. J. Psychiatr. Res..

[101] Gliwińska A., Czubilińska-Łada J., Więckiewicz G., Świętochowska E., Badeński A., Dworak M., Szczepańska M. (2023). The Role of Brain-Derived Neurotrophic Factor (BDNF) in Diagnosis and Treatment of Epilepsy, Depression, Schizophrenia, Anorexia Nervosa and Alzheimer’s Disease as Highly Drug-Resistant Diseases: A Narrative Review. Brain Sci..

[102] Barritault D., Plouët J., Courty J., Courtois Y. (1982). Purification, Characterization, and Biological Properties of the Eye-Derived Growth Factor from Retina: Analogies with Brain-Derived Growth Factor. J. Neurosci. Res..

[103] Wang C.S., Kavalali E.T., Monteggia L.M. (2022). BDNF Signaling in Context: From Synaptic Regulation to Psychiatric Disorders. Cell.

[104] Nibuya M., Morinobu S., Duman R.S. (1995). Regulation of BDNF and TrkB MRNA in Rat Brain by Chronic Electroconvulsive Seizure and Antidepressant Drug Treatments. J. Neurosci..

[105] Sen S., Duman R., Sanacora G. (2008). Serum Brain-Derived Neurotrophic Factor, Depression, and Antidepressant Medications: Meta-Analyses and Implications. Biol. Psychiatry.

[106] Zhou C., Zhong J., Zou B., Fang L., Chen J., Deng X., Zhang L., Zhao X., Qu Z., Lei Y. (2017). Meta-Analyses of Comparative Efficacy of Antidepressant Medications on Peripheral BDNF Concentration in Patients with Depression. PLoS ONE.

[107] De Simone S., Bosco M.A., La Russa R., Vittorio S., Di Fazio N., Neri M., Cipolloni L., Baldari B. (2022). Suicide and Neurotrophin Factors: A Systematic Review of the Correlation between BDNF and GDNF and Self-Killing. Healthcare.

[108] Colucci-D’Amato L., Speranza L., Volpicelli F. (2020). Neurotrophic Factor BDNF, Physiological Functions and Therapeutic Potential in Depression, Neurodegeneration and Brain Cancer. Int. J. Mol. Sci..

[109] Pei Y., Smith A.K., Wang Y., Pan Y., Yang J., Chen Q., Pan W., Bao F., Zhao L., Tie C. (2012). The Brain-Derived Neurotrophic-Factor (BDNF) Val66met Polymorphism Is Associated with Geriatric Depression: A Meta-Analysis. Am. J. Med. Genet. Part B Neuropsychiatr. Genet..

[110] Zhao M., Chen L., Yang J., Han D., Fang D., Qiu X., Yang X., Qiao Z., Ma J., Wang L. (2018). BDNF Val66Met Polymorphism, Life Stress and Depression: A Meta-Analysis of Gene-Environment Interaction. J. Affect. Disord..

[111] Peng Z., Zhou C., Xue S., Bai J., Yu S., Li X., Wang H., Tan Q. (2018). Mechanism of Repetitive Transcranial Magnetic Stimulation for Depression. Shanghai Arch. Psychiatry.

[112] Dieni S., Matsumoto T., Dekkers M., Rauskolb S., Ionescu M.S., Deogracias R., Gundelfinger E.D., Kojima M., Nestel S., Frotscher M. (2012). BDNF and Its Pro-Peptide Are Stored in Presynaptic Dense Core Vesicles in Brain Neurons. J. Cell Biol..

[113] Balkowiec A., Katz D.M. (2002). Cellular Mechanisms Regulating Activity-Dependent Release of Native Brain-Derived Neurotrophic Factor from Hippocampal Neurons. J. Neurosci..

[114] Soppet D., Escandon E., Maragos J., Middlemas D.S., Raid S.W., Blair J., Burton L.E., Stanton B.R., Kaplan D.R., Hunter T. (1991). The Neurotrophic Factors Brain-Derived Neurotrophic Factor and Neurotrophin-3 Are Ligands for the TrkB Tyrosine Kinase Receptor. Cell.

[115] Ji Y., Pang P.T., Feng L., Lu B. (2005). Cyclic AMP Controls BDNF-Induced TrkB Phosphorylation and Dendritic Spine Formation in Mature Hippocampal Neurons. Nat. Neurosci..

[116] Andreska T., Lüningschrör P., Sendtner M. (2020). Regulation of TrkB Cell Surface Expression-a Mechanism for Modulation of Neuronal Responsiveness to Brain-Derived Neurotrophic Factor. Cell Tissue Res..

[117] Minichiello L. (2009). TrkB Signalling Pathways in LTP and Learning. Nat. Rev. Neurosci..

[118] Zakharenko S.S., Patterson S.L., Dragatsis I., Zeitlin S.O., Siegelbaum S.A., Kandel E.R., Morozov A. (2003). Presynaptic BDNF Required for a Presynaptic but Not Postsynaptic Component of LTP at Hippocampal CA1-CA3 Synapses. Neuron.

[119] Zanos P., Thompson S.M., Duman R.S., Zarate C.A., Gould T.D. (2018). Convergent Mechanisms Underlying Rapid Antidepressant Action. CNS Drugs.

[120] Zanos P., Gould T.D. (2018). Mechanisms of Ketamine Action as an Antidepressant. Mol. Psychiatry.

[121] Moghaddam B., Adams B., Verma A., Daly D. (1997). Activation of Glutamatergic Neurotransmission by Ketamine: A Novel Step in the Pathway from NMDA Receptor Blockade to Dopaminergic and Cognitive Disruptions Associated with the Prefrontal Cortex. J. Neurosci..

[122] Chowdhury G.M.I., Zhang J., Thomas M., Banasr M., Ma X., Pittman B., Bristow L., Schaeffer E., Duman R.S., Rothman D.L. (2017). Transiently Increased Glutamate Cycling in Rat PFC Is Associated with Rapid Onset of Antidepressant-like Effects. Mol. Psychiatry.

[123] Jang G., MacIver M.B. (2021). Ketamine Produces a Long-Lasting Enhancement of CA1 Neuron Excitability. Int. J. Mol. Sci..

[124] Zhang B., Yang X., Ye L., Liu R., Ye B., Du W., Shen F., Li Q., Guo F., Liu J. (2021). Ketamine Activated Glutamatergic Neurotransmission by GABAergic Disinhibition in the Medial Prefrontal Cortex. Neuropharmacology.

[125] Falkenberg T., Lindefors N., Camilli F., Metsis M., Ungerstedt U. (1996). Glutamate Release Correlates with Brain-Derived Neurotrophic Factor and TrkB MRNA Expression in the CA1 Region of Rat Hippocampus. Mol. Brain Res..

[126] Derkach V.A., Oh M.C., Guire E.S., Soderling T.R. (2007). Regulatory Mechanisms of AMPA Receptors in Synaptic Plasticity. Nat. Rev. Neurosci..

[127] Henley J.M., Wilkinson K.A. (2016). Synaptic AMPA Receptor Composition in Development, Plasticity and Disease. Nat. Rev. Neurosci..

[128] Pham T.H., Defaix C., Nguyen T.M.L., Mendez-David I., Tritschler L., David D.J., Gardier A.M. (2020). Cortical and Raphe GABAA, AMPA Receptors and Glial GLT-1 Glutamate Transporter Contribute to the Sustained Antidepressant Activity of Ketamine. Pharmacol. Biochem Behav..

[129] Chen Y., Shen M., Liu X., Xu J., Wang C. (2022). The Regulation of Glutamate Transporter 1 in the Rapid Antidepressant-Like Effect of Ketamine in Mice. Front. Behav. Neurosci..

[130] El Iskandrani K.S., Oosterhof C.A., El Mansari M., Blier P. (2015). Impact of Subanesthetic Doses of Ketamine on AMPA-Mediated Responses in Rats: An in Vivo Electrophysiological Study on Monoaminergic and Glutamatergic Neurons. J. Psychopharmacol..

[131] Ma X., Yang S., Zhang Z., Liu L., Shi W., Yang S., Li S., Cai X., Zhou Q. (2022). Rapid and Sustained Restoration of Astrocytic Functions by Ketamine in Depression Model Mice. Biochem. Biophys. Res. Commun..

[132] Koike H., Iijima M., Chaki S. (2011). Involvement of AMPA Receptor in Both the Rapid and Sustained Antidepressant-like Effects of Ketamine in Animal Models of Depression. Behav. Brain Res..

[133] Walker A.K., Budac D.P., Bisulco S., Lee A.W., Smith R.A., Beenders B., Kelley K.W., Dantzer R. (2013). NMDA Receptor Blockade by Ketamine Abrogates Lipopolysaccharide-Induced Depressive-Like Behavior in C57BL/6J Mice. Neuropsychopharmacology.

[134] Björkholm C., Jardemark K., Schilström B., Svensson T.H. (2015). Ketamine-like Effects of a Combination of Olanzapine and Fluoxetine on AMPA and NMDA Receptor-Mediated Transmission in the Medial Prefrontal Cortex of the Rat. Eur. Neuropsychopharmacol..

[135] Zhou W., Wang N., Yang C., Li X.-M., Zhou Z.-Q., Yang J.-J. (2014). Ketamine-Induced Antidepressant Effects Are Associated with AMPA Receptors-Mediated Upregulation of MTOR and BDNF in Rat Hippocampus and Prefrontal Cortex. Eur. Psychiatry.

[136] Qu Y., Shan J., Wang S., Chang L., Pu Y., Wang X., Tan Y., Yamamoto M., Hashimoto K. (2021). Rapid-Acting and Long-Lasting Antidepressant-like Action of (R)-Ketamine in Nrf2 Knock-out Mice: A Role of TrkB Signaling. Eur. Arch. Psychiatry Clin. Neurosci..

[137] Patapoutian A., Reichardt L.F. (2001). Trk Receptors: Mediators of Neurotrophin Action. Curr. Opin. Neurobiol..

[138] Sarbassov D.D., Ali S.M., Sabatini D.M. (2005). Growing Roles for the MTOR Pathway. Curr. Opin. Cell Biol..

[139] Wang J.Q., Mao L. (2019). The ERK Pathway: Molecular Mechanisms and Treatment of Depression. Mol. Neurobiol..

[140] Garro-Martínez E., Fullana M.N., Florensa-Zanuy E., Senserrich J., Paz V., Ruiz-Bronchal E., Adell A., Castro E., Díaz Á., Pazos Á. (2021). MTOR Knockdown in the Infralimbic Cortex Evokes A Depressive-like State in Mouse. Int. J. Mol. Sci..

[141] Li N., Lee B., Liu R.-J., Banasr M., Dwyer J.M., Iwata M., Li X.-Y., Aghajanian G., Duman R.S. (2010). MTOR-Dependent Synapse Formation Underlies the Rapid Antidepressant Effects of NMDA Antagonists. Science.

[142] Abdallah C.G., Averill L.A., Gueorguieva R., Goktas S., Purohit P., Ranganathan M., D’Souza D.C., Formica R., Southwick S.M., Duman R.S. (2018). Rapamycin, an Immunosuppressant and MTORC1 Inhibitor, Triples the Antidepressant Response Rate of Ketamine at 2 Weeks Following Treatment: A Double-Blind, Placebo-Controlled, Cross-over, Randomized Clinical Trial. bioRxiv.

[143] Averill L.A., Averill C.L., Gueorguieva R., Fouda S., Sherif M., Ahn K.-H., Ranganathan M., D’Souza D.C., Southwick S.M., Sanacora G. (2022). MTORC1 Inhibitor Effects on Rapid Ketamine-Induced Reductions in Suicidal Ideation in Patients with Treatment-Resistant Depression. J. Affect Disord..

[144] Nowacka M.M., Obuchowicz E. (2012). Vascular Endothelial Growth Factor (VEGF) and Its Role in the Central Nervous System: A New Element in the Neurotrophic Hypothesis of Antidepressant Drug Action. Neuropeptides.

[145] Hutton C.P., Déry N., Rosa E., Lemon J.A., Rollo C.D., Boreham D.R., Fahnestock M., deCatanzaro D., Wojtowicz J.M., Becker S. (2015). Synergistic Effects of Diet and Exercise on Hippocampal Function in Chronically Stressed Mice. Neuroscience.

[146] Jin K., Zhu Y., Sun Y., Mao X.O., Xie L., Greenberg D.A. (2002). Vascular Endothelial Growth Factor (VEGF) Stimulates Neurogenesis In Vitro and In Vivo. Proc. Natl. Acad. Sci. USA.

[147] Choi M., Lee S.H., Chang H.L., Son H. (2016). Hippocampal VEGF Is Necessary for Antidepressant-like Behaviors but Not Sufficient for Antidepressant-like Effects of Ketamine in Rats. Biochim. Biophys. Acta.

[148] Deyama S., Bang E., Kato T., Li X.-Y., Duman R.S. (2019). Neurotrophic and Antidepressant Actions of Brain-Derived Neurotrophic Factor Require Vascular Endothelial Growth Factor. Biol. Psychiatry.

[149] Deyama S., Bang E., Wohleb E.S., Li X.-Y., Kato T., Gerhard D.M., Dutheil S., Dwyer J.M., Taylor S.R., Picciotto M.R. (2019). Role of Neuronal VEGF Signaling in the Prefrontal Cortex in the Rapid Antidepressant Effects of Ketamine. Am. J. Psychiatry.

[150] Zheng W., Zhou Y.-L., Wang C.-Y., Lan X.-F., Zhang B., Zhou S.-M., Yan S., Yang M.-Z., Nie S., Ning Y.-P. (2021). Association of Plasma VEGF Levels and the Antidepressant Effects of Ketamine in Patients with Depression. Ther. Adv. Psychopharmacol..

[151] Anderson M.F., Åberg M.A.I., Nilsson M., Eriksson P.S. (2002). Insulin-like Growth Factor-I and Neurogenesis in the Adult Mammalian Brain. Dev. Brain Res..

[152] O’Kusky J.R., Ye P., D’Ercole A.J. (2000). Insulin-like Growth Factor-I Promotes Neurogenesis and Synaptogenesis in the Hippocampal Dentate Gyrus during Postnatal Development. J. Neurosci..

[153] Carson M.J., Behringer R.R., Brinster R.L., McMorris F.A. (1993). Insulin-like Growth Factor I Increases Brain Growth and Central Nervous System Myelination in Transgenic Mice. Neuron.

[154] Woods K.A., Camacho-Hübner C., Savage M.O., Clark A.J.L. (1996). Intrauterine Growth Retardation and Postnatal Growth Failure Associated with Deletion of the Insulin-Like Growth Factor I Gene. N. Engl. J. Med..

[155] Beck K.D., Powell-Braxton L., Widmer H.R., Valverde J., Hefti F. (1995). Igf1 Gene Disruption Results in Reduced Brain Size, CNS Hypomyelination, and Loss of Hippocampal Granule and Striatal Parvalbumin-Containing Neurons. Neuron.

[156] Mitschelen M., Yan H., Farley J.A., Warrington J.P., Han S., Hereñú C.B., Csiszar A., Ungvari Z., Bailey-Downs L.C., Bass C.E. (2011). Long-Term Deficiency of Circulating and Hippocampal Insulin-like Growth Factor I Induces Depressive Behavior in Adult Mice: A Potential Model of Geriatric Depression. Neuroscience.

[157] Tu K.-Y., Wu M.-K., Chen Y.-W., Lin P.-Y., Wang H.-Y., Wu C.-K., Tseng P.-T. (2016). Significantly Higher Peripheral Insulin-Like Growth Factor-1 Levels in Patients With Major Depressive Disorder or Bipolar Disorder Than in Healthy Controls. Medicine.

[158] Grunbaum-Novak N., Taler M., Gil-Ad I., Weizman A., Cohen H., Weizman R. (2008). Relationship between Antidepressants and IGF-1 System in the Brain: Possible Role in Cognition. Eur. Neuropsychopharmacol..

[159] Park S.-E., Dantzer R., Kelley K.W., McCusker R.H. (2011). Central Administration of Insulin-like Growth Factor-I Decreases Depressive-like Behavior and Brain Cytokine Expression in Mice. J. Neuroinflammation.

[160] Hoshaw B.A., Malberg J.E., Lucki I. (2005). Central Administration of IGF-I and BDNF Leads to Long-Lasting Antidepressant-like Effects. Brain Res..

[161] Burgdorf J., Zhang X., Colechio E.M., Ghoreishi-Haack N., Gross A., Kroes R.A., Stanton P.K., Moskal J.R. (2015). Insulin-Like Growth Factor I Produces an Antidepressant-Like Effect and Elicits N-Methyl-D-Aspartate Receptor Independent Long-Term Potentiation of Synaptic Transmission in Medial Prefrontal Cortex and Hippocampus. Int. J. NeuroPsychopharmacol..

[162] Deyama S., Kondo M., Shimada S., Kaneda K. (2022). IGF-1 Release in the Medial Prefrontal Cortex Mediates the Rapid and Sustained Antidepressant-like Actions of Ketamine. Transl. Psychiatry.

[163] Carro E., Nuñez A., Busiguina S., Torres-Aleman I. (2000). Circulating Insulin-Like Growth Factor I Mediates Effects of Exercise on the Brain. J. Neurosci..

[164] McMullen E.P., Lee Y., Lipsitz O., Lui L.M.W., Vinberg M., Ho R., Rodrigues N.B., Rosenblat J.D., Cao B., Gill H. (2021). Strategies to Prolong Ketamine’s Efficacy in Adults with Treatment-Resistant Depression. Adv. Ther..

[165] Mithoefer M.C., Feduccia A.A., Jerome L., Mithoefer A., Wagner M., Walsh Z., Hamilton S., Yazar-Klosinski B., Emerson A., Doblin R. (2019). MDMA-Assisted Psychotherapy for Treatment of PTSD: Study Design and Rationale for Phase 3 Trials Based on Pooled Analysis of Six Phase 2 Randomized Controlled Trials. Psychopharmacology.

[166] Jerome L., Feduccia A.A., Wang J.B., Hamilton S., Yazar-Klosinski B., Emerson A., Mithoefer M.C., Doblin R. (2020). Long-Term Follow-up Outcomes of MDMA-Assisted Psychotherapy for Treatment of PTSD: A Longitudinal Pooled Analysis of Six Phase 2 Trials. Psychopharmacology.

[167] Alipoor M., Loripoor M., Kazemi M., Farahbakhsh F., Sarkoohi A. (2021). The Effect of Ketamine on Preventing Postpartum Depression. J. Med. Life.

[168] Han Y., Li P., Miao M., Tao Y., Kang X., Zhang J. (2022). S-Ketamine as an Adjuvant in Patient-Controlled Intravenous Analgesia for Preventing Postpartum Depression: A Randomized Controlled Trial. BMC Anesthesiol..

[169] Monks D.T., Palanisamy A., Jaffer D., Singh P.M., Carter E., Lenze S. (2022). A Randomized Feasibility Pilot-Study of Intravenous and Subcutaneous Administration of Ketamine to Prevent Postpartum Depression after Planned Cesarean Delivery under Neuraxial Anesthesia. BMC Pregnancy Childbirth.

[170] Mohammad Shehata I., Masood W., Nemr N., Anderson A., Bhusal K., Edinoff A.N., Cornett E.M., Kaye A.M., Kaye A.D. (2022). The Possible Application of Ketamine in the Treatment of Depression in Alzheimer’s Disease. Neurol. Int..

[171] Can A.T., Hermens D.F., Dutton M., Gallay C.C., Jensen E., Jones M., Scherman J., Beaudequin D.A., Yang C., Schwenn P.E. (2021). Low Dose Oral Ketamine Treatment in Chronic Suicidality: An Open-Label Pilot Study. Transl. Psychiatry.

[172] Norbury A., Rutter S.B., Collins A.B., Costi S., Jha M.K., Horn S.R., Kautz M., Corniquel M., Collins K.A., Glasgow A.M. (2021). Neuroimaging Correlates and Predictors of Response to Repeated-Dose Intravenous Ketamine in PTSD: Preliminary Evidence. Neuropsychopharmacology.

